# Housing Initiatives to Address Strep A Infections and Reduce RHD Risks in Remote Indigenous Communities in Australia

**DOI:** 10.3390/ijerph21091262

**Published:** 2024-09-23

**Authors:** Nina Lansbury, Paul C. Memmott, Rosemary Wyber, Clarissa Burgen, Samuel K. Barnes, Jessica Daw, Jeffrey Cannon, Asha C. Bowen, Rachel Burgess, Patricia N. Frank, Andrew M. Redmond

**Affiliations:** 1 School of Public Health, Faculty of Medicine, The University of Queensland, Brisbane, QLD 4072, Australia; p.memmott@uq.edu.au (P.C.M.);; 2 Yardhura Walani, National Centre for Epidemiology and Public Health, Australian National University, Canberra, ACT 0200, Australia; 3 The Kids Research Institute, Perth, WA 6009, Australiarachel.burgess@telethonkids.org.au (R.B.); 4 Barkly Region Community, Tennant Creek, NT 0860, Australia; 5 Perth Children’s Hospital, Perth, WA 6009, Australia; 6 Royal Brisbane and Women’s Hospital, The University of Queensland, Brisbane, QLD 4072, Australia

**Keywords:** housing, environmental health, Indigenous health, group A *Streptococcus* (Strep A), rheumatic heart disease, impetigo

## Abstract

Group A *Streptococcus* (Strep A) skin infections (impetigo) can contribute to the development of acute rheumatic fever (ARF) and rheumatic heart disease (RHD). This is of particular concern for Indigenous residents of remote communities, where rates of ARF and RHD are much higher than their urban and non-Indigenous counterparts. There are three main potential Strep A transmission pathways: skin to skin, surface to skin, and transmission through the air (via droplets or aerosols). Despite a lack of scientific certainty, the physical environment may be modified to prevent Strep A transmission through environmental health initiatives in the home, identifying a strong role for housing. This research sought to provide an outline of identified household-level environmental health initiatives to reduce or interrupt Strep A transmission along each of these pathways. The identified initiatives addressed the ability to wash bodies and clothes, to increase social distancing through improving the livability of yard spaces, and to increase ventilation in the home. To assist with future pilots and evaluation, an interactive costing tool was developed against each of these initiatives. If introduced and evaluated to be effective, the environmental health initiatives are likely to also interrupt other hygiene-related infections.

## 1. Introduction

Functional and uncrowded housing can support the health and well-being of residents, including preventing the transmission of hygiene-related infectious diseases related to skin, respiratory, and ear, nose, and throat, among others [1,2]. These preventative health approaches are predominantly enabled through “environmental health” initiatives. Environmental health has long been defined as the prevention of hazards that can undermine human health (Frumkin 2001). In this research, the definition is broadened to incorporate a focus on “salutogenic” (health-supportive) contributions from natural and built environments [3].

The salutogenic nine Healthy Living Practices (HLPs) were developed in Australia in 1987 by a consortium focused on improving the health of remote-living Indigenous Peoples. These describe the essential aspects required in a home to support personal health, including the ability to wash people, wash clothes and bedding, remove waste safely, support nutrition, reduce crowding, and reduce dust [4,5]. Of these, environmental health initiatives can realize the HLPs focused on washing people, clothes, and bedding, removing waste, and reducing crowding and dust [4,5]. Since the establishment of the HLPs, a range of environmental health programs in remote communities have been developed to enable these practices [6,7,8].

Despite the health benefits from and human rights of living on remote Country (Aboriginal and Torres Strait Islander traditional estate) in functional housing and with smaller households to support the health of residents, recent data indicate this is not occurring in many remote and predominantly Indigenous towns and communities in Australia [9,10]. This situation is illustrated by the high burden of *Streptococcus pyogenes* infections (group A *Streptococcus*; herein referred to as “Strep A”) and sequelae, including rheumatic heart disease [11].

Strep A is the primary pathogen of pharyngitis (sore throat) and impetigo (skin sores or “school sores”) in tropical environments, and is the second-most common in temperate regions [10,12,13]. Specifically focusing on skin infections, the world’s highest prevalence of skin sores occurs in Australia’s remote communities [13,14]. One study found that over 80% of children in these communities had had impetigo in their first year of life [15].

Strep A infections of the skin or throat can lead to subsequent conditions (sequelae) such as invasive Strep A infection, post-streptococcal glomerulonephritis (PSGN), chronic kidney disease (CKD), acute rheumatic fever (ARF) and rheumatic heart disease (RHD) [10,16]. RHD is a chronic disease that can limit life quality and length [17]. The burden of each of these sequelae is greatest for Aboriginal and Torres Strait Islander people, particularly in Northern Australia [11].

The disproportionate burden of Strep A-associated disease for Indigenous Peoples, namely, RHD, is unlikely to be driven by genetics, ethnicity, or geographic location, noting that early last century, there were high rates of RHD across Australia, including in urban and non-Indigenous populations [18]. The incidence of RHD among non-Indigenous people declined from the 1950s in parallel with improvements in environmental and socio-economic factors that have reduced exposure to and transmission of Strep A. These factors include smaller households and availability of functioning health hardware such as functional, well-maintained plumbing and washing machines [18].

Despite these urban health improvements, in Australia, RHD persists in many remote Indigenous towns and communities. These settlements are often characterized by lower employment and income levels, crowded housing, reduced access to culturally safe medical care (through remoteness and distance from health-care and service providers), and a lack of access to adequate and functioning health infrastructure [19,20,21]. The consolidation of these risk factors for Strep A infection and subsequent ARF is a consequence of colonization and systemic racism [22,23]. Risks may be modifiable through environmental health initiatives as a form of primordial prevention [5,16]

Such efforts would align with the Australian government’s stated Priority 7 in the National Aboriginal and Torres Strait Islander Health Plan (2021–2031) to focus on prevention through “healthy environments.” This aims to eliminate RHD by 2031 [24]. This need was described by one of the paper’s three Aboriginal authors who is based in a remote community:

*We need a Wumperarni [local Indigenous Peoples’] checklist for staff to take house to house to check up and advise on life skills to prevent illness. Wumperarni staff should do the visits- male plus female- because this avoids shame... Whitefellas can be bossy, dictate the rules and be judgmental. The checklist would be in plain English and ask about how many people live here? How many children and dogs are there? Who is immunized? Is there cleaning equipment like a bucket, mop, broom, toilet brush and cleaning products? Then the clinic can organize appointments for health checks or make public health referrals*

(Patricia Narrurlu Frank, personal communication, 4 July 2024).

Existing scientific understanding about Strep A, including transmission pathways, is the subject of active ongoing research, and review and re-analysis of historic studies has provided recent new insights [21,25,26], as have international outbreak responses [27].

While further research is underway, Indigenous communities are taking action to prevent Strep A infections using the most pragmatic, evidence-informed approaches currently available [7,28]. Aligned with such community-led approaches, this research has been conducted employing the precautionary principle—seeking to identify options to act preventatively when human health (or the environment) is threatened [29].

Using the precautionary approach, interruption opportunities were sought in this research that addressed one of three Strep A transmission pathways likely to make the most substantive contribution in Aboriginal and Torres Strait Islander contexts. The first pathway is direct contact, termed “skin to skin” in this paper. The likelihood of Strep A transmission via skin-to-skin contact has been identified since the 1930s and is particularly associated with Strep A skin infections [30,31]. The second pathway is through fomites, termed “surface to skin” here. This pathway is where Strep A transmission may occur via contact with hard surfaces [30] or soft materials, such as bedding, towels, and clothing [32]. The third pathway is transmission through the air of Strep A via droplets or aerosols, and may involve dust as a fomite [25,27,30,31]. Various publications describe the impact of desiccation of Strep A transmission through the air from extremes of temperatures [33]. Rapid desiccation of cells is likely to significantly reduce Strep A viability before re-infection can occur, though this effect may be modified by environmental factors such as droplet composition, temperature, and humidity [34,35,36]. More definitive evidence is required to understand the effect of these variables in real world settings.

Strep A transmission pathways may be interrupted through environmental health initiatives, especially those that realize one or more HLPs, which are grounded in the broad priorities for healthy living in remote Indigenous communities [1,37]. Therefore, this research sought to provide an outline of identified and costed environmental health initiatives that may limit the transmission of Strep A. In doing so, Strep A and other hygiene-related infections may be reduced in addition to preventing the development of later chronic diseases, including RHD.

## 2. Methods

The methods applied to identify possible environmental health initiatives that may reduce transmission of Strep A in remote community homes and then to calculate approximate costs for these involved identifying the main transmission pathways of Strep A in housing through a narrative review, identifying which built environment aspects (“environmental health initiatives”) could interrupt Strep A transmission pathways, and identifying the cost of the identified environmental health initiatives through an analysis of actual financial costings.

### 2.1. Positioning of the Research

This research is contextualized in remote Australia and recognizes and respects Aboriginal and Torres Strait Islander Australians who wish to remain living on Country as the custodians, irrespective of remoteness from major urban centers. It also acknowledges this as a human right under the United Nations (UN) Declaration on the Rights of Indigenous Peoples [38]. This declaration recognizes that living on ancestral Country can provide health-supportive outcomes in terms of social, emotional, spiritual, and physical well-being [39]. From herein, the term “Indigenous Peoples” is used, unless more specific identity is known, to refer respectfully to Australia’s First Peoples, variously known as Aboriginal and Torres Strait Islander Peoples and First Nations Peoples [40].

### 2.2. Key Definitions

Strep A transmission is defined here as the movement of the Strep A bacteria from one source to the subsequent acquisition by a human recipient, with a focus on superficial skin and throat infections (rather than invasive Strep A disease that follows either of these transmission pathways) [41]. The outcome of this acquisition may or may not result in a Strep A-related infection, depending on bacterial load of the colonization, efficacy of the transmission, route of transmission, and susceptibility of the recipient [42].

### 2.3. Identifying Environmental Health Initiatives to Interrupt Strep A Transmission

A narrative review [43] was adopted as the method to locate potential environmental initiatives that may reduce or disrupt the three likely transmission pathways of Strep A bacteria (skin to skin, surface to skin, transmission through the air). Health databases (PubMed, Medline, and Web of Science) were searched for full-text publications from the past twelve years (2012–2024). The search employed the keywords of environmental health, intervention, Strep*, and transmission. Duplications were removed, then abstracts reviewed, and appropriate papers selected.

Additionally, publications were provided by the project’s disciplinary community of practice members that consisted of researchers from a variety of organizations with expertise in infectious diseases medicine, architecture, anthropology, and environmental health [44]. These members shared relevant publications including “gray literature” published by government and non-government organizations, in addition to specific academic publications. This formed a version of “manual searching” as a complementary approach to database searches to produce a more comprehensive review of relevant publications [45].

### 2.4. Costing Identified Initiatives

A Microsoft Excel tool was created to estimate the indicative costs of, and enable comparison between the identified environmental health initiatives (see Supplementary File). The tool’s development was subject to a number of outlined assumptions. This resulting tool provides a benchmark for broad budgeting requirements. Users can adapt and refine this tool for their specific circumstance.

Cost fields were included for capital infrastructure, including housing and “health hardware” such as washing machines and hot water systems; repairs and maintenance; training and educational activities; materials and consumables, including water and electricity consumption; and miscellaneous items. The costs within each environmental health initiative are interactive, including a variety of options from which the user can choose. Users can input community-specific demographic factors, such as population size, number of dwellings, and remoteness index [46]. The remoteness indicator is a multiplier that adjusts construction and other costs based on different geographical locations. It is based on combined data from the 2022 Rawlinson’s *Australian Construction Handbook* as applied by the National Disability Insurance Agency, which was in turn informed by actual build costs from both government and private sector projects [46,47].

Resulting costs are summarized in total (i.e., societal perspective), and stratified by payer (community members, local government, local housing organization, or other) and payment frequency (once off or ongoing). Costs are estimated in Australian dollars at 2022–2023 price levels. It is important to note that these are only indicative costs. The geographic location of implementation would involve specific costing circumstances (particularly concerning separation by payer), and this will require further community and stakeholder consultation, possibly in combination with an evaluation of existing environmental health infrastructure. Furthermore, the tool does not account for any existing infrastructure. Instead, all infrastructure is considered as a new installation.

## 3. Results

The resulting information was organized by pathways of transmission, and subsequently by the prevention outcome that was sought. This outcome was aligned with an associated HLP where relevant. Specific environmental health initiatives were then described that met this outcome, HLP, and pathway. Each initiative was described by the type within the categories that emerged, namely, physical, behavioral, health promotion, financial, or human resources. These are summarized in Table 1.

### 3.1. Preventing Skin-to-Skin Strep A Transmission

#### 3.1.1. Cleaning Harmful Bacteria from the Skin in the Home (1.1)

The 2020 Australian Guideline for RHD [17] identifies a “strong association” between the ability to wash hands and bodies and the reduction in the risk of Strep A infections. This is based on international randomized controlled trials where daily handwashing with soap and water was associated with a reduction in impetigo (alongside other infectious diseases) [48].

An environmental health initiative to support healthy skin is to ensure the maintenance of health hardware that enables Healthy Living Practice 1, notably provision of a functioning shower, available hot water, non-medicated soap supplies or body wash [49]. Extensive surveys of health hardware functionality and immediate repairs have been successfully conducted in remote communities since the late 1980s under the Housing for Health program that seeks to provide the ability to practice all nine Healthy Living Practices [5]. Where implemented in New South Wales and central Australia, there has been a reduced incidence of hygiene-related infectious diseases [8,19].

The effect of this health hardware initiative can be amplified through integrating environmental health referrals to other health service engagements such as clinic visits for repeated skin infections and family support visits. This has been an innovation by some health services whereby local environmental health workers and officers create a link between their own communities and clinical services [50,51].

#### 3.1.2. Cleaning Harmful Bacteria from the Skin Outside the Home (1.2)

Community swimming pools (especially those that require showering with non-medicated soap and shampoo before swimming) may provide a mechanism outside of the home environment for reducing bacterial load on the skin through both showering and the presence of chlorine in the pool water [16,49,52]. The most common age group of users is school-aged children, where the highest burden of skin sores also occurs [13]. A systematic review identified a decline in prevalence and severity of skin infections after opening a swimming pool or implementing a community-based swimming program [52].

The availability of the swimming pool initiative is limited by requiring trained lifeguards as well as the expense of construction. An alternative is to provide a “water park” with water fountains, but not swimming facilities [53]. Both swimming and water park options are limited by being used in specific seasons with hot weather, the existence of the facility, water treatment, requirement for repairs and maintenance, and preference by certain age groups (noting that babies and older people may not use the facilities as frequently) [53].

#### 3.1.3. Covering Skin Infections and Wound Care to Prevent Bacterial Transmission and Heal Damaged Skin (1.3)

Wound care and wound covering are complementary health approaches to home-based initiatives that reduce the microbial load in the local environment as well as potentially reducing skin-to-skin transmission [54]. In hot and humid conditions, some occlusive dressings may lead to sores becoming excessively macerated, so tailored care to optimize wound healing and reduce transmission is beneficial [55].

Existing and previous initiatives involving home visits have been noted to increase presentations to clinics and to ultimately reduce the incidence of skin sores [50,56]. These can identify and treat wounds, encourage clinic visits, and provide first aid kits to homes as well as instructions for use [49,50,56]. These actions may shift the attention placed on skin sore infections from being “normalized” by patients and health-care workers to being a focus and of concern [57].

#### 3.1.4. Social Distancing in Home through Additional Housing and Rooms (1.4)

There is a strong relationship between the prevalence of skin sores and household crowding [10,58]. The burden of Strep A, ARF infections, and RHD have been noted to be up to 2.8 times higher in crowded households [16]. A contemporary case–control study in New Zealand found a strong association between household crowding and ARF risk (OR 3.88, 95% CI 1.68–8.69) [59].

There has been recent emphasis on prevention of transmission of Strep A infections through reducing crowding [16]. Specific approaches include increasing available housing stock, enhancing new housing design to facilitate social distancing between householders, increasing floor space of existing housing stock and ensuring repair and maintenance of existing housing stock, including health hardware functionality [32,37,56].

#### 3.1.5. Social Distancing in Home through Outdoor Living Spaces (Livable Yard and Veranda) (1.5)

Expanding living spaces in existing houses can enable opportunities for social distancing and for hosting visitors without resulting in unintended crowding within the home, and thus could reduce the risk of Strep A transmission [17]. For example, expanded sitting and sleeping spaces can be achieved through large verandas with shade and insect screening, outdoor fans, outdoor misting lines, and yard-based shade structures and bough sheds [60]. In addition, landscaping initiatives such as shade trees and lawn could provide cooler and additional living spaces [61].

Post-construction, this initiative would require behavioral changes to use the yard for socializing and sleeping and health promotion to emphasize the value of social distancing to reduce infection transmission.

#### 3.1.6. Social Distancing When Sleeping (1.6)

Co-sleeping with more than one person per bed in close contact may enable Strep A transmission through both skin-to-skin contact [32] and transmission through air or droplets [30]. In response, enabling social distancing when sleeping could likely reduce Strep A transmission [56]. This could be supported through physical initiatives, such as providing additional bedding (mattresses and bed frames), and potentially avoid co-sleeping, particularly if people have skin sores or sore throats [32].

However, while co-sleeping may be a result of limited beds due to limited financial means, it can also be a preferred arrangement for ensuring a sense of security, safety, warmth, and cultural traditions [62]. Therefore, reconsidering co-sleeping requires discussion with communities for cultural understanding and sensitivity [32].

### 3.2. Preventing Surface-to-Skin Strep A Transmission

#### 3.2.1. Ability to Clean Bed Linen, Towels, Clothing (2.1)

The risk of bacterial transmission and associated infections may be increased when households have limited access to washing and drying facilities for clothing and bedding (including blankets) [1]. This has been shown in the pathogen *Staphylococcus aureus* [63].

This might be a plausible intervention that affects fomites in Strep A transmission [31] and/or when the material is heavily contaminated with excretions from skin infections or infected nasal discharge [17].

Disinfection of linen involves access to laundry facilities for washing and drying, hot water supplies, laundry detergent, and washing lines (including pegs), in addition to spare sets of linen and towels. Laundry facilities can be achieved through functioning washing machines in the home (with access to tradespeople and parts for repairs), a mobile laundry service, or a community-based laundry facility [64,65]. Laundry detergent supplies need to be available and affordable [32]. Sufficient power supply and availability of dryers (such as through community facilities) are required, noting that power supply must be prepaid in many remote Australian communities, posing ongoing challenge with security of household energy supply [66]. Community ownership, accessibility, and acceptability could be coupled with key health messages to support regular use of laundry facilities.

#### 3.2.2. Ability to Clean Hard Surfaces (2.2)

Strep A has been identified as causing infection from skin contact with contaminated surfaces in institutional settings [31,67,68]. For example, a Strep A outbreak in a military institution showed high loads of bacteria transmitted directly and indirectly through contact with gym equipment (ropes and benches) and room dust [67]. An outbreak of Strep A in a nursing home showed high bacterial loads on carpets and furnishings (upholstered chairs and curtains) [68].

Environmental contamination with Strep A can potentially be reduced by cleaning activities [69]. Although it is not yet clear in the literature whether disinfection or cleaning has the most influential role in interrupting bacterial transmission, it is expected that transmission can be reduced through access to cleaning products and regular use of these products on surfaces that are in direct contact with householders. Culturally responsive training programs to enhance living skills in a built environment might support this kind of health promotion, such as the (now discontinued) Homemaker program in Central Australia [70]. However, there is no locatable evidence base about the effects of different cleaning products or an evaluation of the Homemaker program [32]. Household studies to confirm the need for this activity are needed, as evidence from health-care settings is currently being extrapolated.

### 3.3. Preventing Strep A Transmission through the Air

One initiative to reduce transmission of Strep A through the air is described under 1.6 “Social distancing when sleeping (avoid co-sleeping).” Two other initiatives are described below.

#### Ventilation of the Home (3.1)

Ventilation has been demonstrated to reduce transmission of diseases in aerosol form [71]. Of note, increased awareness and subsequent responses to improve ventilation and cross-ventilation, especially in hospital settings, rapidly increased during the COVID-19 pandemic in response to airborne transmission events [72]. Some newly emerging evidence indicates that this may also apply for Strep A, raising the potential for ventilation to be explored as a risk reduction strategy [35]. Further research is needed to explore this possible but untested hypothesis.

Ventilation of the home could occur through physical initiatives including installation of fans and adjustable windows (with insect screens) or air purifiers, although it is worth noting that household occupancy, design, and climate affect how such an initiative can be effectively installed [72]. These aspects and evidence indicate a need for further investigation regarding both levels of home ventilation prior to any corresponding ventilation initiative, such as using a carbon dioxide monitor to measure air-exchange rate [73]. Different modalities will be needed depending on the relative contribution of droplet and aerosol transmission. Whilst hospital and laboratory studies have been conducted, no home-based publications are available to enhance understanding of these modalities of transmission.

### 3.4. Estimation of Costs Using New Costings Tool

**Figure 1:**
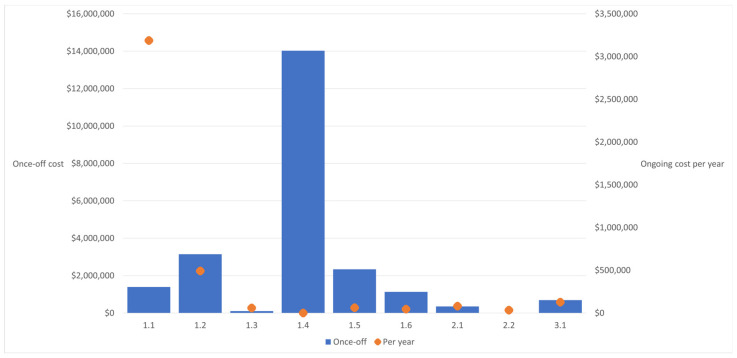
Cost estimates (upfront and ongoing) of each of the environmental health initiatives for a model remote Indigenous community in Northern Territory, Australia.

To provide indicative cost estimates of delivering each of the identified environmental health initiatives (EHIs), the costings tool was modeled using an example of a remote Indigenous town in the Northern Territory, Australia. It was assumed to have 10 people per household [2], with 100 households in the town. Indicative costs for this model town can be viewed in Figure 1. Further details with the stratification of each sub-category (e.g., physical, behavioral, and health promotion), as well as assumed payer (person/organization likely to bear burden of the cost), can be found in the Supplementary File.

For the model remote town, the upfront once-off costs ranged from <AUD 10,000 (EHI 2.2) to just over AUD 14 million (EHI 1.4). Of the nine environmental health initiatives, the upfront once-off costs for two were <AUD 200,000 (EHI 1.3 and 2.2); two were between AUD 200,000 and 1 million (EHI 2.1 and 3.1); and four were between AUD 1 and 4 million (EHI 1.1, 1.2, 1.5 and 1.6); and one was >AUD 14 million (EHI 1.4). The ongoing annual costs ranged from AUD 0 per year (EHI 1.4) to just over AUD 3.1 million per year (EHI 1.1). Of the nine environmental health initiatives, the ongoing annual costs for one was AUD 0 (EHI 1.4); five were <AUD 100,000 (EHI 1.3, 1.5, 1.6, 2.1 and 2.2); two were between AUD 100,000 and AUD 500,000 (EHI 1.2 and 3.1); and one was >AUD 500,000 (EHI 1.1).

## 4. Discussion and Conclusions

This research focused on three potential transmission pathways for Strep A and identified ten potential prevention outcomes that addressed four healthy living practices. These outcomes included a range of environmental health initiatives of relevance to remote Indigenous community homes and that involve physical, behavioral, health promotion, financial, and human resources. Indicative costings using an interactive tool developed for this research were calculated.

The contributions of this paper are threefold: to outline the potential contribution of environmental health initiatives in reducing Strep A transmission, to identify possible initiatives to pilot with evaluation frameworks, and to provide a costings tool for tailoring and application to a range of settings. The costings tool is anticipated to be of relevance for use by environmental health officers and workers, housing organizations, and health-care providers seeking to prevent Strep A and other hygiene-related infections and associated diseases if these mechanisms are proven scientifically to be effective.

Limitations of this research included the paucity of published evidence on the efficacy of each initiative, further reinforcing the recommendation for a thorough review of such initiatives prior to adoption. This research was also limited by uncertainty on the relative contribution of each of the potential Strep A transmission pathways, noting that review work is underway [25]. Further limitations are the feasibility of implementing some or all of the initiatives in a comprehensive and sustainable manner in remote communities where financial costs for maintenance and repair are high, environmental conditions for infrastructure are harsh, and available trade services for repairs and maintenance are limited. Therefore, any trial of initiatives will require dedicated ongoing resources to support ongoing health improvements.

The current limited understanding of Strep A transmission interruption would be strengthened through prospective data collection with contemporary methodology to clearly define the drivers of Strep A transmission in remote community settings. Such research would ideally enable a community-led selection of initiatives to pilot and subsequent evaluate how Strep A can be reduced. Evaluation would require an appropriate tool to be developed. It could consider the Aboriginal definition of health and well-being, changes in infection rates, genomic information of Strep A infections, cultural and local acceptance of initiatives, cost–benefit analysis (including maximum coverage of household residents), and available, sufficient, and sustainable funding of initiatives.

This research is pertinent in Australia, where decades of research and government reports have documented insufficient housing stock for the population size in remote communities and a lack of regular and acute repair and maintenance. This has led to crowding and limitations to the ability to undertake healthy living practices to maintain the health of householders. This situation is counter to upholding the human rights of Indigenous Peoples living on their traditional Country [38].

This research is also relevant internationally, as Strep A infections and sequelae have declined since the 1940s in high-income settings, broadly due to smaller households, availability of antibiotics for bacterial infections, and an increase in the standard of living, with parallel increases in financial resources. Thus, for populations that continue to have high burdens of Strep A infection, ARF, and RHD, the need remains urgent to consider the living context in terms of financial, political, and physical aspects.

## Figures and Tables

**Table 1:** Environmental health initiatives to potentially reduce Strep A transmission in household settings.

Strep A Transmission Pathway Prevention Outcome Sought		Type of Initiative (Physical, Behavioral, Health Promotion, Financial, Human Resources)	Healthy Living Practice Alignment (9 Practices)
1. Skin to skin			
	1.1. Cleaning harmful bacteria from the skin in the home	Physical (functioning shower, functioning hot water, non-medicated soap supplies; body wash) Behavioral (hand and face washing, daily showering, use of soap supplies) Human resources (environmental health referrals from health clinic and family services)	1. Washing people
	1.2. Cleaning harmful bacteria from the skin in community pool/water park facilities	Physical (installation of community pool or water park) Behavioral (attending and using water park or pool; showering with soap before entering) Financial (cost of maintaining community pool or water park; ongoing water treatment) Human resources (trained lifeguards)	1. Washing people
	1.3. Covering skin infections, and wound care to prevent bacterial transmission and heal damaged skin	Physical (first aid supplies) Behavioral (covering wounds) Health promotion (recommendation for wound care); Financial (funding for health worker to visit, identify and dress wounds)	No alignment
	1.4. Social distancing in home through additional housing and rooms	Physical (building and renovations) Behavioral (sleeping arrangements) Health promotion (improve health literacy on infection transmission) Financial (furnishings for the larger space)	5. Reducing crowding
	1.5. Social distancing in home through outdoor living spaces (livable yard and veranda)	Physical (landscaping; outdoor shade; outdoor fan) Behavioral (use of yard for social interactions, guests’ sleeping space, maintenance with yard cleaning and repairs) Health promotion (improve health literacy on infection transmission) Financial (additional outdoor ablution/bathroom facilities)	5. Reducing crowding
	1.6. Social distancing when sleeping	Physical (extra mattresses, bedding, and bed frames) Behavioral (avoid co-sleeping; increase frequency of washing bed linen)	5. Reducing crowding
2. Surface to skin			
	2.1. Ability to clean bed linen, towels, clothing	Physical (working washing machine in the home/mobile laundry service/community laundry facility; laundry detergent supplies; washing line and pegs; spare sets of linen; available dryer) Behavioral (regular changing, washing and drying of sheets/blankets, linen, towels, clothes) Health promotion (supporting uptake of washing and drying facilities) Financial (mattress replacement, washing machine and dryer, second set of linen and clothes, etc.)	2. Washing clothes and bedding
	2.2. Ability to clean hard surfaces	Physical (cleaning products, wiping cloths) Behavioral (use of cleaning products) Health promotion (explaining and encouraging disinfection) Financial (cost of cleaning supplies)	n/a
3. Through air to the respiratory tract			
	3.1. Ventilation of the home	Physical (installation of fans, air conditioner, adjustable windows, flyscreens) Behavioral (cough etiquette; use of ventilation) Health promotion (use of ventilation)	n/a
	(see also 1.6 Social distancing when sleeping	Physical (extra mattresses, bedding, and bed frames) Behavioral (avoid co-sleeping; increase frequency of washing bed linen)	5. Reducing crowding

## Data Availability

Costing data are available in the Supplementary File.

## Supplementary Materials

The following supporting information can be downloaded at https://www.mdpi.com/article/10.3390/ijerph21091262/s1. Supplementary File: Costing tool template (attached).
